# Design and Synthesis of Highly Active Antimycobacterial Mutual Esters of 2-(2-Isonicotinoylhydrazineylidene)propanoic Acid

**DOI:** 10.3390/ph14121302

**Published:** 2021-12-14

**Authors:** Václav Pflégr, Jana Maixnerová, Jiřina Stolaříková, Adrián Pál, Jana Korduláková, František Trejtnar, Jarmila Vinšová, Martin Krátký

**Affiliations:** 1 Department of Organic and Bioorganic Chemistry, Faculty of Pharmacy in Hradec Králové, Charles University, Akademika Heyrovského 1203, 500 05 Hradec Králové, Czech Republic; pflegrv@faf.cuni.cz (V.P.); vinsova@faf.cuni.cz (J.V.); 2 Department of Pharmacology and Toxicology, Faculty of Pharmacy in Hradec Králové, Charles University, Akademika Heyrovského 1203, 500 05 Hradec Králové, Czech Republic; maixj6a1@faf.cuni.cz (J.M.); trejtnarf@faf.cuni.cz (F.T.); 3 Laboratory for Mycobacterial Diagnostics and Tuberculosis, Regional Institute of Public Health in Ostrava, Partyzánské náměstí 7, 702 00 Ostrava, Czech Republic; Jirina.Stolarikova@zuova.cz; 4 Department of Biochemistry, Faculty of Natural Sciences, Comenius University in Bratislava, Mlynská dolina CH-1, Ilkovičova 6, 842 15 Bratislava, Slovakia; pal9@uniba.sk (A.P.); jana.kordulakova@uniba.sk (J.K.)

**Keywords:** antimycobacterial activity, enzyme inhibition, esters, InhA, isoniazid, mechanism of action, mutual prodrugs

## Abstract

The combination of two active scaffolds into one molecule represents a proven approach in drug design to overcome microbial drug resistance. We designed and synthesized more lipophilic esters of 2-(2-isonicotinoylhydrazineylidene)propanoic acid, obtained from antitubercular drug isoniazid, with various alcohols, phenols and thiols, including several drugs, using carbodiimide-mediated coupling. Nineteen new esters were evaluated as potential antimycobacterial agents against drug-sensitive *Mycobacterium tuberculosis* (*Mtb*.) H_37_Rv, *Mycobacterium avium* and *Mycobacterium kansasii*. Selected derivatives were also tested for inhibition of multidrug-resistant (MDR) *Mtb*., and their mechanism of action was investigated. The esters exhibited high activity against *Mtb*. (minimum inhibitory concentrations, MIC, from ≤0.125 μM), *M. kansasii*, *M. avium* as well as MDR strains (MIC from 0.25, 32 and 8 µM, respectively). The most active mutual derivatives were derived from 4-chloro/phenoxy-phenols, triclosan, quinolin-8-ol, naphthols and terpene alcohols. The experiments identified enoyl-acyl carrier protein reductase (InhA), and thus mycobacterial cell wall biosynthesis, as the main target of the molecules that are activated by KatG, but for some compounds can also be expected adjunctive mechanism(s). Generally, the mutual esters have also avoided cytotoxicity and are promising hits for the discovery of antimycobacterial drugs with improved properties compared to parent isoniazid.

## 1. Introduction

Currently, tuberculosis (TB) and COVID-19 are worldwide pandemic infectious diseases that cause the most deaths in the world. Both diseases have similar symptoms such as cough, fever and difficulty breathing. However, TB has a longer incubation period with a slower onset of disease [1]. About one-quarter of the world’s population is estimated to be infected with *Mycobacterium tuberculosis* (*Mtb*.), the bacteria that give rise to the disease, especially in low-resource countries including regions with high poverty, lacking basic amenities and medical facilities [2]. Anti-TB medicines have been used for decades and strains that are resistant to one or more of the medicines have been documented in every country surveyed. Worldwide, only 57% of multidrug-resistant (MDR) TB patients are currently successfully treated. MDR-TB remains resistant to isoniazid (INH) and rifampicin (RIF) [3], the most effective anti-TB drugs.

One of the oldest and specific drugs for tuberculosis treatment is INH, having bactericidal effect on active and bacteriostatic effect on inactive stages of *Mtb*. INH is a prodrug, which needs to be activated by the multifunctional catalase peroxidase enzyme KatG into a range of activated species, such as an isonicotinoyl radical, which can acylate numerous compounds. It forms a complex with nicotinamide adenine dinucleotide (NAD^+^), which inhibits NADH-dependent enoyl-ACP (acyl carrier protein) reductase (InhA) and thus prevents elongation of the mycolic acid chain. This is considered primary and the main mechanism of action. However, the activation of INH also produces various oxygen species radicals that disrupt the synthesis of deoxyribonucleic acid (DNA), lipids, carbohydrates, and NAD^+^ [4]. A long duration of treatment leads to the resistance to INH, which is most often associated with the mutations in *katG*, *inhA* and, to a lesser extent, several other genes (e.g., *ndh*, *kasA* and oxyReahpC intergenic region) [5,6].

INH remains a key component of all multiple regimens recommended by the WHO, even if resistant isolates develop rapidly, especially during monotherapy or inappropriate treatment [7]. Improving INH by introducing chemical modifications into its basic structure in order to enhance the biological response to *Mtb*. and/or circumvent resistance remains an interesting scientific challenge [8,9]. Pyridine core of INH was modified without finding a molecule of higher activity, but the modification of the hydrazide part can offer derivatives with improved pharmacological profile [10].

It is believed that the hydrazone functional group increases the lipophilicity of the parent compounds (hydrazines and hydrazides), which results in an enhancement of transport through microbial membranes and cell walls [11].

Inspired by these facts and continuing our ongoing research in the field of the synthesis and antimicrobial activity of medicinally important antituberculotics, the synthesis of some novel “me-too” derivatives of INH as esters of (*E*)-2-(2-isonicotinoylhydrazineylidene)propanoic acid are reported. A molecular hybridization approach was employed. We designed and prepared mutual esters mainly with bioactive alcohols, phenols, and thiols, including established drugs, which have predominantly their own antimicrobial activity. Thus, in addition to higher lipophilicity, the proposed mutual antimycobacterial esters offer the potential benefits of dual antimycobacterial activity, additivity/synergism and also combating drug-resistant pathogens due to their different mechanisms of action.

Bifunctional pyruvic acid easily forms a hydrazone bond with INH, and free carboxyl can be esterified. Esters are the most common prodrug strategies used to improve the lipophilicity [12,13]. This kind of mutual antibacterial esters of pyrazinoic acid with substituted salicylanilides demonstrated an effective and promising treatment against pathogenic fungi and bacteria (especially against Gram-positive, tuberculous, and atypical mycobacterial strains), including INH-resistant mycobacteria, which are resistant to one or more drugs used clinically [13]. INH-based hydrazone acid amides have also been described as highly active agents against TB [14]. In addition, pyridine-based compounds have been extensively investigated as potential antibacterial, antimycobacterial, antifungal and anticancer drugs [15,16,17,18].

## 2. Materials and Methods

### 2.1. Chemistry

#### 2.1.1. General

All chemicals for synthesis and analysis were purchased from Merck KGaA (Darmstadt, Germany), Penta Chemicals Unlimited (Prague, Czech Republic) or Lach-Ner (Neratovice, Czech Republic) and were used as received without further purification. The structures of the prepared substances were confirmed via ^1^H NMR and ^13^C NMR spectroscopy analysis. NMR spectra (500 MHz for ^1^H and 126 MHz for ^13^C) were measured in DMSO-*d_6_*, DMF-*d_7_* or CDCl_3_ as solvents at ambient temperature by a Varian VNMR S500 instrument (Varian, Palo Alto, CA, USA). The values of chemical shifts (δ) are given in parts per million (ppm) and the spectra were referenced internally, to tetramethylsilane as standard by the residual signal of protic solvent (DMSO-*d_6_*: 2.50 for ^1^H, 39.70 for ^13^C; DMF-*d_7_*: 2.75, 2.92, 8.03 for ^1^H, 29.76, 34.89, 163.15 for ^13^C and CDCl_3_: 7.25 for ^1^H, 77.10 for ^13^C). The coupling constants (*J*) are reported in Hz. Infrared spectra were recorded by a Nicolet 6700 FT-IR spectrometer (Thermo Fisher Scientific, Waltham, MA, USA) in the range 600–4000 cm^−1^; ATR (Ge) technique of measuring was used. Elemental analysis was performed on a Vario MICRO Cube Element Analyzer (Elementar Analysensysteme, Hanau, Germany). Both calculated and found values are given as percentages. Melting points were recorded using a Büchi B-545 apparatus (BÜCHI Labortechnik AG, Flawil, Switzerland). Retention factors (*R_f_*) of all prepared compounds, and reaction progresses were analysed by thin layer chromatography (TLC); the plates were coated with 0.2 mm Merck 60 F254 silica gel (Merck Millipore, Darmstadt, Germany) and were visualised by UV irradiation (254 nm). Dichloromethane/methanol (S1) (4:0.3 *v*/*v*) and dichloromethane/acetone (S2) (4:0.5 *v*/*v*) mixtures were used as the eluents for TLC and for column chromatography. Merck Kieselgel 60 Å (0.040–0.063 mm) Merck KGaA (Darmstadt, Germany) was used for column chromatography. 

The calculated log*P* values (Clog*P*), which are the logarithms of the partition coefficients for octan-1-ol/water and reaction schemes, were acquired using the program ChemDraw Professional 18.1 (PerkinElmer Inc., Waltham, MA, USA).

#### 2.1.2. Synthesis

Procedure for synthesis of (*E*)-2-(2-isonicotinoylhydrazineylidene)propanoic acid [14,19] (**2**)

Isoniazid (274.3 mg, 2.0 mmol) was dissolved in hot methanol (15 mL) with stirring; pyruvic acid (193.7 mg, 155 μL, 2.2 mmol) was then added dropwise. The reaction mixture was refluxed for 2 h, then let to cool at room temperature and stored for 1 h at 8 °C. Precipitate was filtered off and washed with methanol (2 × 3 mL). Yield: 98% of (*E*)-isomer as a white solid; mp: 181.1–183.0 °C. IR (ATR): 660, 753, 787, 859, 894, 925, 1048, 1107, 1161, 1246, 1271, 1322, 1374 (CH_3_), 1430, 1481, 1521, 1541, 1691 (CONH), 2342, 2359, 3045, 3101 (N-H), 3502 cm^−1^. ^1^H NMR (500 MHz, DMSO-*d_6_*): δ 13.59 (1H, s, COOH), 11.06 (1H, s, CONH), 8.83–8.69 (2H, m, H2, H6), 7.79–7.67 (2H, m, H3, H5), 2.14 (3H, s, CH_3_). ^13^C NMR (126 MHz, DMSO): δ 166.05, 164.64, 150.93, 150.47, 150.10, 121.11, 20.68. Elemental analysis; calculated: C, 52.17; H, 4.38; N, 20.28, found: C, 52.24; H, 4.14; N, 20.29. R*_f_* (S1): 0.20.

General procedure for the synthesis of (*E*)-2-(2-isonicotinoylhydrazineylidene)propanoic acid esters (**3a**–**3s**)

(*E*)-2-(2-Isonicotinoylhydrazineylidene)propanoic acid (207.2 mg, 1.0 mmol), 4-(dimethylamino)pyridine (DMAP; 12.2 mg, 0.1 mmol) and appropriate alcohol or thiol (1.1 mmol) were suspended in 35 mL of dichloromethane under stirring. The reaction mixture was cooled to 0 °C, then 1.3 of equivalents of *N*-(3-dimethylaminopropyl)-*N*′-ethylcarbodiimide hydrochloride (EDC.HCl; 249.2 mg, 1.3 mmol) was added in one portion. The reaction mixture was allowed to react at 0 °C for 4 h, followed by 12 h (overnight) stirring at room temperature. The volume of the reaction mixture was reduced to 15 mL under reduced pressure, transferred to a separatory funnel and washed with 10% solution of sodium carbonate (2 × 15 mL) and 15 mL of saturated brine. The organic layer was separated and dried with anhydrous sodium sulphate. The solvent was evaporated under reduced pressure, and the product was purified via crystallization using various solvents or column chromatography.

Methyl (*E*)-2-(2-isonicotinoylhydrazineylidene)propanoate [20] (**3a**). White solid; yield 90%; mp 122.0–124.8 °C. IR (ATR): 634, 652, 681, 705, 754, 776, 832, 1109, 1151, 1175, 1300, 1366, 1384 (CH_3_), 1408, 1552, 1622, 1678 (CONH), 1723 (COO), 2973, 3183 (N-H) cm^−1^. ^1^H NMR (500 MHz, DMSO-*d*_6_): δ 11.16 (1H, s, CONHN), 8.80–8.72 (2H, m, H2, H6), 7.80–7.72 (2H, m, H3, H5), 3.75 (3H, s, OCH_3_), 2.17 (3H, s, CH_3_). ^13^C NMR (126 MHz, DMSO): δ 165.08, 163.68, 150.95, 150.20, 140.77, 122.41, 52.64, 13.66. Elemental analysis; calculated: C, 54.30; H, 5.01; N, 19.00, found: C, 54.35; H, 5.00; N, 19.00. Purification by column chromatography using S1 as eluent; R*_f_*: 0.25.

Phenyl (*E*)-2-(2-isonicotinoylhydrazineylidene)propanoate (**3b**). White solid; yield 58%; mp 186.0–189.0 °C. IR (ATR): 622, 659, 725, 835, 1134, 1163, 1190, 1212, 1271, 1373 (CH_3_), 1506, 1537, 1689 (CONH), 1729 (COO), 3042, 3210 (N-H) cm^−1^. ^1^H NMR (500 MHz, DMF-*d_7_*): δ 11.60 (1H, s, CONHN), 8.99–8.95 (2H, m, H2, H6), 8.07–8.04 (2H, m, H3, H5), 7.67 (2H, t, *J* = 7.9 Hz, H3′, H5’), 7.50 (1H, t, *J* = 7.4 Hz, H4’), 7.44 (2H, d, *J* = 8.0 Hz, H2’, H6’), 2.59 (3H, s, CH_3_). ^13^C NMR (126 MHz, DMF): δ 164.13, 162.55, 151.87, 150.83, 150.29, 141.46, 130.23, 126.63, 122.39, 13.26. Elemental analysis; calculated: C, 63.60; H, 4.63; N, 14.83, found: C, 63.52; H, 4.21; N, 19.99. Purification by column chromatography using S1 as eluent; R*_f_*: 0.31.

*p*-Tolyl (*E*)-2-(2-isonicotinoylhydrazineylidene)propanoate (**3c**). White solid; yield 65%; mp 168.0–170.0 °C. IR (ATR): 617, 660, 725, 804, 1135, 1165, 1194, 1271, 1372 (CH_3_), 1410, 1508, 1538, 1688 (CONH), 1730 (COO), 3043, 3209 (N-H) cm^−1^. ^1^H NMR (500 MHz, CDCl_3_): δ 9.71 (1H, s, CONHN), 8.81–8.76 (2H, m, H2, H6), 7.91–7.85 (2H, m, H3, H5), 7.20 (2H, d, *J* = 8.3 Hz, H3’, H5’), 7.02 (2H, d, *J* = 8.4 Hz, H2’, H6’), 2.36 (3H, s, Ph-CH_3_), 2.32 (3H, s, CH_3_). ^13^C NMR (126 MHz, CDCl_3_): δ 162.96, 160.77, 150.05, 148.43, 139.13, 135.88, 130.01, 129.99, 124.20, 120.91, 20.85, 11.53. Elemental analysis; calculated: C, 64.64; H, 5.09; N, 14.13, found: C, 64.66; H, 5.00; N, 14.01. Purification by column chromatography using S1 as eluent; R*_f_*: 0.33.

4-Methoxyphenyl (*E*)-2-(2-isonicotinoylhydrazineylidene)propanoate (**3d**). Yellow solid; yield 52%; mp 169.0–172.0 °C. IR (ATR): 617, 636, 660, 699, 726, 753, 820, 837, 1138, 1160, 1192, 1252, 1273, 1372 (CH_3_), 1412, 1439, 1506, 1535, 1688 (CONH), 1729 (COO), 3239 (N-H) cm^−1^. ^1^H NMR (500 MHz, CDCl_3_): δ 9.81 (1H, s, CONHN), 8.79–8.76 (2H, m, H2, H6), 7.92–7.85 (2H, m, H3, H5), 7.07–7.03 (2H, m, H2’, H6’), 6.92–6.88 (2H, m, H3’, H5’), 3.80 (3H, s, OCH_3_), 2.23 (3H, s, CH_3_). ^13^C NMR (126 MHz, CDCl_3_): δ 164.56, 163.11, 157.47, 150.18, 150.01, 144.12, 139.39, 124.12, 122.01, 114.51, 55.57, 11.70. Elemental analysis; calculated: C, 61.34; H, 4.83; N, 13.41, found: C, 61.44; H, 5.02; N, 13.49. Purification by column chromatography using S1 as eluent; R*_f_*: 0.27.

4-Phenoxyphenyl (*E*)-2-(2-isonicotinoylhydrazineylidene)propanoate (**3e**). White solid; yield: 60%; mp 152.4–155.0 °C. IR (ATR): 604, 617, 651, 636, 665, 690, 722, 741, 769, 838, 900, 1071, 1094, 1127, 1140, 1185, 1206, 1258, 1375 (CH_3_), 1487, 1501, 1662, 1673 (CONH), 1754 (COO), 3039, 3260 (N-H) cm^−1^. ^1^H NMR (500 MHz, DMSO-*d*_6_): δ 11.42 (1H, s, CONHN), 8.80–8.74 (2H, m, H2, H6), 7.81–7.76 (2H, m, H3, H5), 7.43–7.39 (2H, m, H3”, H5”), 7.26–7.14 (3H, H3’, H5’, H4”), 7.09–7.02 (4H, H2’, H6’, H2”, H6”), 2.27 (3H, s, CH_3_). ^13^C NMR (126 MHz, DMSO): δ 163.26, 161.90, 156.80, 154.50, 150.18, 146.24, 140.70, 130.29, 123.81, 123.33, 122.49, 119.60, 118.83, 13.66. Elemental analysis; calculated: C, 67.19; H, 4.56; N, 11.19, found: C, 67.24; H, 4.62; N, 11.09. Crystallization from tetrahydrofuran/*n-*hexane. R*_f_* (S1): 0.32.

4-Fluorophenyl (*E*)-2-(2-isonicotinoylhydrazineylidene)propanoate (**3f**). White solid; yield 68%; mp: 174.1–177.2 °C. IR (ATR): 665, 694, 706, 729, 762, 816, 827, 839, 874, 1016, 1068, 1089, 1129, 1138, 1191, 1252, 1269, 1376 (CH_3_), 1495, 1505, 1530, 1660, 1671 (CONH), 1751 (COO) cm^−1^. ^1^H NMR (500 MHz, DMSO-*d*_6_): δ 11.38 (1H, s, CONHN), 8.80–8.73 (2H, m, H2, H6), 7.80–7.75 (2H, m, H3, H5), 7.32–7.23 (4H, m, H2’, H3’, H5’, H6’), 2.26 (3H, s, CH_3_). ^13^C NMR (126 MHz, DMSO): δ 164.02, 163.34, 159.87 (d, *J* = 241.8 Hz), 150.16, 149.90, 146.86 (d, *J* = 2.8 Hz), 140.63, 123.69 (d, *J* = 8.7 Hz), 122.49, 116.38 (d, *J* = 23.4 Hz), 13.64. Elemental analysis; calculated: C, 59.80; H, 4.01; N, 13.95, found: C, 59.77; H, 4.10; N, 14.00. Crystallization from ethyl acetate/*n-*hexane. R*_f_* (S1): 0.30.

4-Chlorophenyl (*E*)-2-(2-isonicotinoylhydrazineylidene)propanoate (**3g**). White solid; yield 55%, mp: 184.0–186.1 °C. IR: 634, 561, 722, 741, 751, 837, 887, 1015, 1086, 1140, 1153, 1167, 1206, 1286, 1293, 1384 (CH_3_), 1409, 1490, 1621, 1681 (CONH), 1716, 1736 (COO), 3101, 3182 (N-H), 3566 cm^−1^. ^1^H NMR (500 MHz, DMSO-*d*_6_): δ 11.44 (1H, s, CONHN), 8.78–8.73 (2H, m, H2, H6), 7.81–7.76 (2H, m, H3, H5), 7.51 (2H, d, *J* = 8.4 Hz, H3’, H5’), 7.26 (2H, d, *J* = 8.2 Hz, H2’, H6’), 2.26 (3H, s, CH_3_). ^13^C NMR (126 MHz, DMSO): δ 161.55, 154.50, 150.44, 149.90, 143.26, 140.30, 130.61, 129.99, 124.16, 123.89, 13.69. Elemental analysis; calculated: C, 56.70; H, 3.81; N, 13.23, found: C, 56.66; H, 3.99; N, 13.29. Crystallization from ethyl acetate/*n-*hexane. R*_f_* (S1): 0.31.

4-Bromophenyl (*E*)-2-(2-isonicotinoylhydrazineylidene)propanoate (**3h**). White solid; yield 92%; mp: 190.0–192.0 °C. IR (ATR): 632, 718, 751, 837, 1068, 1141, 1154, 1168, 1203, 1293, 1387 (CH_3_), 1483, 1682 (CONH), 1734 (COO), 3083, 3182 (N-H) cm^−1^. ^1^H NMR (500 MHz, DMSO-*d*_6_): δ 11.36 (1H, s, CONHN), 8.81–8.74 (2H, m, H2, H6), 7.82–7.74 (2H, m, H3, H5), 7.64 (2H, d, *J* = 8.4 Hz, H3’, H5’), 7.20 (2H, d, *J* = 8.4 Hz, H2’, H6’), 2.26 (3H, s, CH_3_). ^13^C NMR (126 MHz, DMSO): δ 163.07, 157.51, 150.17, 150.08, 140.73, 140.62, 132.66, 124.28, 122.49, 118.53, 13.65. Elemental analysis; calculated: C, 49.74; H, 3.34; N, 11.60, found: C, 49.70; H, 3.39; N, 11.59. Crystallization from benzene. R*_f_* (S1): 0.31.

Naphthalen-1-yl (*E*)-2-(2-isonicotinoylhydrazineylidene)propanoate (**3i**). White solid; yield 58%; mp: 181.1–182.5 °C. IR (ATR): 609, 663, 706, 748, 769, 777, 796, 804, 1077, 1123, 1161, 1223, 1259, 1377 (CH_3_), 1485, 1512, 1693 (CONH), 1715 (COO), 3292 (N-H) cm^−1^. ^1^H NMR (500 MHz, DMSO-*d*_6_): δ 11.44 (1H, s, CONHN), 8.81–8.72 (2H, m, H2, H6), 8.01 (1H, d, *J* = 7.9 Hz, H8’), 7.92–7.72 (4H, m, H3, H5, H4’, H5’), 7.62–7.52 (3H, m, H3’, H6’, H7’), 7.41 (1H, d, *J* = 7.5 Hz, H2’), 2.34 (3H, s, CH_3_). ^13^C NMR (126 MHz, DMSO): δ 163.44, 157.56, 150.22, 149.72, 146.42, 140.65, 134.32, 128.15, 126.95, 126.90, 126.34, 126.16, 125.90, 122.49, 121.15, 118.46, 13.82. Elemental analysis; calculated: C, 68.46; H, 4.54; N, 12.61, found: C, 68.53; H, 4.61; N, 12.66. Purification by column chromatography using S2 as eluent; R*_f_*: 0.16.

Naphthalen-2-yl (*E*)-2-(2-isonicotinoylhydrazineylidene)propanoate (**3j**). White solid; yield 73%; mp: 116.0–117.2 °C. IR (ATR): 633, 646, 688, 754, 819, 843, 870, 1079, 1120, 1141, 1177, 1211, 1244, 1273, 1290, 1342, 1359 (CH_3_), 1410, 1513, 1597, 1672 (CONH), 1707 (COO), 3230 (N-H) cm^−1^. ^1^H NMR (500 MHz, DMF-*d*_7_): δ 11.47 (1H, s, CONHN), 8.81 (2H, d, *J* = 5.0 Hz, H2, H6), 8.07–7.97 (3H, m, H4’, H5’, H8’), 7.90 (2H, d, *J* = 5.2 Hz, H3, H5), 7.81 (1H, d, *J* = 2.4 Hz, H1’), 7.61–7.53 (2H, m, H6’, H7’), 7.45 (1H, dd, *J* = 7.6, 2.4 Hz, H3’), 2.45 (3H, s, CH_3_). ^13^C NMR (126 MHz, DMF): δ 163.90, 150.62, 150.43, 149.13, 141.76, 141.04, 134.08, 131.79, 129.74, 128.06, 127.86, 127.00, 126.15, 122.57, 121.68, 118.80, 12.95. Elemental analysis; calculated: C, 68.46; H, 4.54; N, 12.61, found: C, 68.55; H, 4.50; N, 12.68. Purification by column chromatography using S2 as eluent; R*_f_*: 0.16.

5-Isopropyl-2-methylphenyl (*E*)-2-(2-isonicotinoylhydrazineylidene)propanoate (**3k**). Colourless foamy solid; yield 81%. IR (ATR): 620, 637, 665, 754, 839, 1068, 1133, 1169, 1240, 1265, 1377 (CH_3_), 1408, 1506, 1531, 1681 (CONH), 1739 (COO), 2961, 3240 (N-H) cm^−1^. ^1^H NMR (500 MHz, CDCl_3_): δ 13.41 (1H, s, CONHN), 8.74–8.71 (2H, m, H2, H6), 7.71–7.68 (2H, m, H3, H5), 7.20 (1H, d, *J* = 7.8 Hz, H3’), 7.09 (1H, dd, *J* = 7.9, 1.8 Hz, H4’), 6.93 (1H, d, *J* = 1.8 Hz, H6’), 2.89 (1H, sept, *J* = 6.9 Hz, CH), 2.15 (3H, s, CH_3_), 2.13 (3H, s, CH_3_), 1.23 (6H, d, *J* = 7.4 Hz, C-CH_3_). ^13^C NMR (126 MHz, CDCl_3_): δ 161.79, 161.33, 150.69, 148.54, 147.88, 139.11, 138.41, 131.17, 126.34, 125.19, 120.90, 118.91, 33.41, 23.69, 20.40, 15.62. Elemental analysis; calculated: C, 67.24; H, 6.24; N, 12.38, found: C, 67.31; H, 6.30; N, 12.39. Purification by column chromatography using S2 as eluent; R*_f_*: 0.32.

2-Isopropyl-5-methyphenyl (*E*)-2-(2-isonicotinoylhydrazineylidene)propanoate (**3l**). Colourless foamy solid; yield 52%. IR (ATR): 605, 616, 638, 646, 562, 664, 684, 704, 731, 744, 755, 820, 840, 896, 919, 1060, 1084, 1132, 1151, 1240, 1271, 1378 (CH_3_), 1409, 1507, 1536, 1620, 1674 (CONH), 1736 (COO), 2966, 3194 (N-H) cm^−1^. ^1^H NMR (500 MHz, DMSO-*d*_6_): δ 11.34 (1H, s, CONHN), 8.80–8.73 (2H, m, H2, H6), 7.81–7.74 (2H, m, H3, H5), 7.24 (1H, d, *J* = 7.9 Hz, H3’), 7.06 (1H, d, *J* = 7.9 Hz, H4’), 6.91 (1H, s, H6’), 2.94–2.84 (1H, m, CH), 2.29 (3H, s, CH_3_), 2.26 (3H, s, CH_3_), 1.11 (6H, d, *J* = 7.1 Hz, C-CH_3_). ^13^C NMR (126 MHz, DMSO): δ 163.50, 150.30, 150.05, 147.90, 145.61, 140.66, 136.76, 136.41, 127.24, 126.93, 126.63, 122.76, 122.48, 26.76, 22.93, 20.50, 13.71. Elemental analysis; calculated: C, 67.24; H, 6.24; N, 12.38, found: C, 67.20; H, 6.31; N, 12.41. Purification by column chromatography using S2 as eluent; R*_f_*: 0.34.

Quinolin-8-yl (*E*)-2-(2-isonicotinoylhydrazineylidene)propanoate (**3m**). Green solid; yield 66%, mp: 159.3–160.4 °C. IR (ATR): 615, 636, 654, 663, 711, 758, 785, 819, 843, 1082, 1130, 1234, 1273, 1370 (CH_3_), 1499, 1540, 1672, 1688 (CONH), 1738 (COO), 3039, 3210 (N-H) cm^−1^. ^1^H NMR (500 MHz, DMSO-*d*_6_): δ 11.39 (1H, s, CONHN), 8.90 (1H, dd, *J* = 4.2, 1.7 Hz, H2’), 8.81–8.73 (2H, m, H2, H6), 8.47 (1H, dd, *J* = 8.3, 1.7 Hz, H4’), 7.96–7.93 (1H, m, H5’), 7.82 (2H, d, *J* = 4.7 Hz, H3, H5), 7.69–7.59 (3H, m, H3’, H6’, H7’), 2.35 (3H, s, CH_3_). ^13^C NMR (126 MHz, DMSO): δ 163.29, 150.85, 150.33, 150.22, 147.28, 140.66, 140.45, 140.34, 136.48, 129.34, 126.65, 126.52, 122.51, 122.38, 121.64, 13.77. Elemental analysis; calculated: C, 64.67; H, 4.22; N, 16.76, found: C, 64.70; H, 4.16; N, 16.80. Crystallization from ethyl acetate/*n-*hexane. R*_f_* (S1): 0.25.

Pyridin-4-ylmethyl (*E*)-2-(2-isonicotinoylhydrazineylidene)propanoate (**3n**). White solid; yield 65%; mp: 126.0–128.0 °C. IR (ATR): 628, 638, 652, 771, 806, 844, 927, 1069, 1078, 1141, 1184, 1265, 1293, 1384 (CH_3_), 1415, 1489, 1512, 1687 (CONH), 1708 (COO), 3268 (N-H), 3566, 3648 cm^−1^. ^1^H NMR (500 MHz, DMSO-*d*_6_): δ 12.83 (1H, s, CONHN), 8.81–8.78 (2H, m, H2, H6), 8.61–8.58 (2H, m, H2’, H6’), 7.70–7.66 (2H, m, H3, H5), 7.47–7.44 (2H, m, H3’, H5’), 5.37 (2H, s, CH_2_), 2.26 (3H, s, CH_3_). ^13^C NMR (126 MHz, DMSO): δ 163.11, 161.77, 150.95, 149.99, 144.14, 140.90, 139.81, 122.15, 121.14, 65.43, 20.41. Elemental analysis; calculated: C, 60.40; H, 4.73; N, 18.78, found: C, 60.30; H, 4.68; N, 18.84. Crystallization from water/acetone. R*_f_* (S1): 0.31.

5-Chloro-2-(2,4-dichlorophenoxy)phenyl (*E*)-2-(2-isonicotinoylhydrazineylidene)propanoate (**3o**). White solid; yield 36%; mp: 153.0–155.0 °C. IR (ATR): 662, 691, 751, 758, 811, 826, 840, 866, 1059, 1067, 1079, 1115, 1125, 1181, 1215, 1267, 1381 (CH_3_), 1408, 1476, 1537, 1670 (CONH), 1751 (COO), 3044, 3232 (N-H) cm^−1^. ^1^H NMR (500 MHz, DMSO-*d*_6_): δ 11.39 (1H, s, CONHN), 8.79–8.71 (2H, m, H2, H6), 7.78–7.56 (4H, m, H3, H5, H6’, H3”), 7.42–7.37 (2H, m, H4’, H5”), 7.15–6.98 (2H, m, H3’, H6”), 2.19 (3H, s, CH_3_). ^13^C NMR (126 MHz, DMSO): δ 166.71, 162.20, 150.66, 150.17, 150.05, 146.46, 141.74, 140.45, 130.19, 129.03, 128.93, 128.39, 127.69, 124.61, 122.84, 122.51, 121.09, 120.94, 13.42. Elemental analysis; calculated: C, 52.69; H, 2.95; N, 8.78, found: C, 52.70; H, 3.00; N, 8.89. Crystallization from water/acetone. R*_f_* (S1): 0.24.

4-Acetamidophenyl 2-(2-isonicotinoylhydrazineylidene)propanoate (**3p**). White solid; yield 82% (mixture of isomers). IR (ATR): 651, 686, 726, 757, 765, 828, 1133, 1155, 1190, 1285, 1344, 1380 (CH_3_), 1408, 1509, 1531, 1589, 1666, 1674, 1687 (CONH), 1723 (COO), 3053, 3221 (N-H), 3301 cm^−1^. ^1^H NMR (500 MHz, DMSO-*d*_6_): δ 12.87 (1H, s, CONHN), 10.05 (1H, s, NHCO), 8.80–8.77 (2H, m, H2, H6), 7.72–7.68 (2H, m, H3, H5), 7.67–7.63 (2H, m, H3’, H5’), 7.24–7.20 (2H, m, H2’, H6’), 2.08 (3H, s, CH_3_), 2.05 (3H, s, CH_3_) (dominant (*E*)-isomer). ^13^C NMR (126 MHz, DMSO): δ 168.53, 167.66, 160.84, 151.77, 150.99, 144.78, 139.98, 137.83, 121.90, 120.03, 115.15, 24.12, 20.59 (dominant (*E*)-isomer). Elemental analysis; calculated: C, 60.00; H, 4.74; N, 16.46, found: C, 59.99; H, 4.80; N, 16.50. The crude product was purified by crystallization from diethyl ether. The compound was prepared as a mixture of geometric isomers *E:Z* = 4:1, which were not further separated. R*_f_* (S1): 0.35 for *E* and 0.31 for *Z.*

2-(2-Methyl-5-nitro-1*H*-imidazol-1-yl)ethyl (*E*)-2-(2 isonicotinoylhydrazineylidene)propanoate (**3q**). Green solid; yield 72%, mp: 123.0–125.7 °C. IR (ATR): 620, 681, 753, 770, 867, 1078, 1147, 1183, 1260, 1268, 1294, 1361 (CH_3_), 1380 (NO_2_), 1432, 1464, 1483, 1512, 1529, 1596 (NO_2_), 1698 (CONH), 1705 (COO), 3265 (N-H) cm^−1^. ^1^H NMR (500 MHz, DMSO-*d*_6_): δ 12.79 (1H, s, CONHN), 8.83–8.78 (2H, m, H2, H6), 8.06 (1H, s, H4’), 7.69–7.65 (2H, m, H3, H5), 4.72 (2H, t, *J* = 5.1 Hz, OCH_2_), 4.57 (2H, t, *J* = 5.1 Hz, NCH_2_), 2.49 (3H, s, imidazole CH_3_), 2.17 (3H, s, CH_3_). ^13^C NMR (126 MHz, DMSO): δ 161.85, 161.31, 151.81, 150.96, 140.37, 139.72, 138.75, 133.47, 121.12, 64.23, 44.29, 20.10, 14.18. Elemental analysis; calculated: C, 50.00; H, 4.48; N, 23.32, found: C, 50.02; H, 4.41; N, 23.35. Crystallization from ethanol/water. R*_f_* (S1): 0.30.

*S*-Ethyl (*E*)-2-(2-isonicotinoylhydrazineylidene)propanethioate (**3r**). White solid; yield 77%, mp: 158.0–160.0 °C. IR (ATR): 639, 665, 729, 752, 772, 787, 834, 919, 1027, 1154, 1249, 1376 (CH_3_), 1407, 1553, 1622, 1671 (COS), 1698 (CONH), 2957, 3066, 3182 (N-H) cm^−1^. ^1^H NMR (500 MHz, CDCl_3_): δ 9.90 (1H, s, CONHN), 8.83–8.80 (2H, m, H2, H6), 7.84–7.78 (2H, m, H3, H5), 2.90 (2H, q, *J* = 7.4 Hz, CH_2_), 2.19 (3H, s, CH_3_), 1.26 (3H, t, *J* = 7.4 Hz, CH_3_). ^13^C NMR (126 MHz, CDCl_3_): δ 191.57, 168.71, 149.90, 146.84, 139.12, 124.00, 22.98, 14.44, 10.08. Elemental analysis; calculated: C, 52.57; H, 5.21; N, 16.72, found: C, 52.64; H, 5.30; N, 16.79. Crystallization from acetone/water. R*_f_* (S1): 0.42.

*S*-Phenyl (*E*)-2-(2-isonicotinoylhydrazineylidene)propanethioate (**3s**). White solid; yield 72%, mp: 186.0–187.0 °C. IR (ATR): 606, 637, 653, 747, 845, 871, 936, 1026, 1147, 1243, 1292, 1373 (CH_3_), 1518, 1676 (COS), 1698 (CONH), 3246 (N-H), 3286 cm^−1^. ^1^H NMR (500 MHz, DMSO-*d*_6_): δ 11.72 (1H, s, CONHN), 8.79 (2H, d, *J* = 4.9 Hz, H2, H6), 7.76 (2H, d, *J* = 4.9 Hz, H3, H5), 7.49–7.36 (5H, m, H2’, H3’, H4’, H5’, H6’), 2.14 (3H, s, CH_3_). ^13^C NMR (126 MHz, DMSO): δ 190.06, 170.11, 150.07, 145.53, 140.65, 135.08, 129.52, 129.37, 128.06, 122.60, 12.01. Elemental analysis; calculated: C, 60.19; H, 4.38; N, 14.04, found: C, 60.22; H, 4.45; N, 14.15. Crystallization from benzene. R*_f_* (S1): 0.40.

### 2.2. Biological Activity

#### 2.2.1. Antimycobacterial Activity

The in vitro antimycobacterial activity of the INH derivatives **3a**–**3s** was evaluated using a previously published method [14]. The panel of mycobacteria comprised one drug-susceptible *Mycobacterium tuberculosis* strain 331/88 (i.e., H_37_Rv; dilution of this strain was 10^−3^) and two strains of nontuberculous mycobacteria: *Mycobacterium avium* 330/88 (resistant to INH, rifamycines, ofloxacin and ethambutol; dilution of the strain was 10^−5^) and a clinical isolate of *Mycobacterium kansasii* (6509/96; dilution of the strain was 10^−4^). The micromethod for the determination of minimum inhibitory concentration (MIC) was used involving the Šula’s semisynthetic medium (SEVAC, Prague, Czech Republic). The investigated compounds were added to the medium as solutions in DMSO; the final volume contained 1.0% DMSO (*v*/*v*). The following concentrations were used: 1000, 500, 250, 125, 62.5, 32, 16, 8, 4, 2, 1, 0.5, 0.25 and 0.125 μM. The MIC were determined after incubation at 37 °C for 14 and 21 days, for *M. kansasii* additionally for 7 days. MIC (in μM) was the lowest concentration at which the complete inhibition of mycobacterial growth occurred. Parent INH was involved as a reference drug.

The most active derivatives (**3d**, **3h**, **3i**, **3j**, **3k**, **3m**, **3o**, **3p**, **3q** and **3s**) were evaluated against seven drug-resistant TB strains (dilution of these strains was 10^−3^) with different resistance patterns. All of the strains are resistant to INH, RIF, rifabutin and streptomycin, and an additional resistance to other drugs was present in some cases: strain 7357/1998 was resistant additionally to ethambutol and ofloxacin; strain 234/2005 to ethambutol; strain 8666/2010 resistant to ethambutol, ofloxacin and clofazimine; strain Praha 1 exhibited an additional resistance to ethambutol and clofazimine; Praha 4 to ethambutol, ofloxacin and clofazimine (all MDR-TB strains); and Praha 131 was resistant to INH, rifamycines, streptomycin, ethambutol, ofloxacin, gentamicin and amikacin (i.e., XDR-TB strain). The following concentrations were used for drug-resistant strains: 1000, 500, 250, 125, 62.5, 32, 16, 8, 4, 2, 1 and 0.5 μM.

##### Analysis of Sensitivity of *Mtb*. H_37_Ra Strains Overproducing InhA and KatG

InhA protein was overproduced in *Mtb*. H_37_Ra using pMV261-*inhA* construct as previously described [21]. KatG protein encoded by the gene *MSMEG_6384* from *Mycobacterium smegmatis* was overproduced in *Mtb*. H_37_Ra using pVV16-*katG_smeg_* construct previously described [14].

Susceptibility of *Mtb*. H_37_Ra strains overproducing InhA_tb_ or KatG_smeg_, and control strains carrying empty pMV261 or pVV16 vectors, to the esters **3l**, **3m** and **3o** was evaluated using the drop dilution method. The cultures grown in 7H9 broth supplemented with albumin–dextrose–catalase and 0.05% Tween 80 were adjusted to optical density (OD)_600_ ~0.5 and 4 µL aliquots of 1, 10^−1^ and 10^−2^ dilutions of the compounds **3** were dropped on 7H11 agar supplemented with oleic acid–albumin–dextrose–catalase and various concentrations of the tested compounds dissolved in DMSO (2% final concentration). Plates were incubated for 28 days at 37 °C.

##### Investigation of mechanism of action

The mode of action was analysed by metabolic labelling of *Mtb*. H_37_Ra strain with ^14^C acetate. *Mtb*. H_37_Ra culture was grown in 7H9 broth supplemented with 10% albumin–dextrose–catalase and 0.05% Tween 80 at 37 °C until OD_600_ reached 0.48. The tested compounds were dissolved in DMSO and added in two final concentrations corresponding to their 1× and 5× MIC values for *Mtb*. H_37_Rv, namely 0.25 and 1.25 µM for **3l** and **3m** and 0.125 and 0.625 µM for **3o**. The final concentration of DMSO was kept at 2%. INH in final concentration of 3.5 µM was used as a control agent. ^14^C acetate (specific activity 110 mCi/mmol, ARC) was added to each of the cultures (in final concentration 0.5 µCi/mL) after 24 h of cultivation with inhibitors and the cells were cultivated for next 24 h.

The lipids from the cells grown in 100 µL cultures were extracted with 1.5 mL chloroform/methanol (1:2, *v*/*v*) at 56 °C for 1 h, followed by the extraction with 1.5 mL chloroform/methanol (2:1, *v*/*v*) at 56 °C for 1.5 h. The organic extracts were combined, dried under N_2_ and subjected to biphasic Folch washing [22]. Isolated lipids were dissolved in 50 μL of chloroform/methanol (2:1, *v*/*v*) and 10 μL was loaded on TLC silica gel plates F254 (Merck). Lipids were separated in chloroform/methanol/water mixture (20:4:0.5, *v*/*v*/*v*) and visualized using an Amersham^TM^ Typhoon^TM^ Biomolecular Imager or by autoradiography.

Fatty acid methyl esters (FAME) and mycolic acids methyl esters (MAME) were prepared from whole cells from 100 µL cultures as previously described [23]. Dried extracts were dissolved in 50 µL of chloroform/methanol (2:1, *v*/*v*) and 10 μL was loaded on TLC plates. FAME and different forms of MAME were separated by three runs in *n*-hexane/ethyl acetate (95:5, 3 runs) and visualized as described for lipids.

#### 2.2.2. Cytotoxic Activity

The human hepatocellular liver carcinoma cell line HepG2 (ATCC HB-8065) purchased from Health Protection Agency Culture Collections (ECACC, Salisbury, UK; passage 8–12) was cultured in Minimum Essentials Eagle Medium (Sigma-Aldrich, St. Louis, MO, USA) supplemented with 10% foetal bovine serum, 1% L-glutamine solution (Sigma-Aldrich) and nonessential amino acid solution (Sigma-Aldrich) in a humidified atmosphere containing 5% CO_2_ at 37 °C.

For subculturing, the cells were harvested after trypsin/EDTA (Sigma-Aldrich) treatment at 37 °C. To determine cytotoxicity of the compounds, the cells treated with the tested substances were used as experimental groups whereas untreated HepG2 cells served as control groups.

The cells were seeded in a density 15,000 cells per well in a 96-well plate. The next day the cells were treated with each of the tested substances at a broad range of concentrations (1–1000 µM) in triplicates; the compounds were dissolved in DMSO (maximal incubation concentration of DMSO was 1% *v*/*v*). The controls representing 100% cell viability, 0% cell viability (the cells treated with 10% DMSO), no cell control and vehiculum controls were incubated in parallel, also as triplicates. After 24 h of incubation in a humidified atmosphere containing 5% CO_2_ at 37 °C, the reagent from the kit CellTiter 96 AQueous One Solution Cell Proliferation Assay (CellTiter 96; PROMEGA, Fitchburg, WI, USA) was added. After 2 h incubation at 37 °C, absorbance of samples was recorded at 490 nm (TECAN, Infinita M200, Grödig, Austria). A standard toxicological parameter IC_50_ was calculated by nonlinear regression from a semilogarithmic plot of incubation concentration versus percentage of absorbance relative to untreated controls using GraphPad Prism 8 software (GraphPad Software, Inc., La Jolla, CA, USA). 

Results of the experiments are presented as inhibitory concentrations that reduce viability of the cell population to 50% from the maximal viability (IC_50_). Parent INH was involved as a reference agent.

## 3. Results and Discussion

### 3.1. Design of Esters

To synthesize highly active antimycobacterial (thio)esters, we used hydrazone acid derived from INH (**1**) and pyruvic acid, 2-(2-isonicotinoylhydrazineylidene)propanoic acid (**2**), which has been shown as an antimycobacterial agent not inferior to parent INH [14].

Regarding alcohols and thiols, mostly bioactive compounds with a known antimicrobial activity were chosen to form mutual derivatives, aiming to obtain molecules with an improved inhibition of mycobacteria. Methanol was used for the synthesis of reference ester **3a**. Phenol (substrate for **3b**) was first used as an antiseptic by Joseph Lister during implementation of antiseptic surgery methodology [24]. It was later described that a mixture of three positional isomers of cresol (tricresol) excels in its antiseptic effects. Owing to these and its other important biological properties, phenol has become an interesting synthetic template for several biologically active compounds, especially antibacterial chemotherapeutics [24]. In general, antimicrobial properties are shared by various phenols, and their halogenation or substitution by lipophilic groups (e.g., alkyl(oxy), aryl(oxy), halogen) boosts their activity (i.e., precursors of **3c**–**3j**) [25]. Monoterpene phenolic derivatives of cymene such as carvacrol (**3k**) and thymol (precursor of **3l**) have also been investigated for their potential antituberculotic properties. Andrade-Ochoa et al. [26] reported very good antimycobacterial activity of essential oils containing carvacrol and thymol. Their extensive QSAR and in vitro studies confirmed that these terpenes may be suitable candidates for antituberculotics. Quinoline and its derivatives are also in the forefront of interest as model structures for the design of antituberculotics (ester **3m**). Thus, of particular interest is the discovery of bedaquiline, an innovative drug approved for the treatment of MDR-TB [27]. Darby et al. [28] described the antituberculotic activity of quinolin-8-ol against latent (nonreplicating) as well as active growing *Mtb*. They point out the properties of quinolin-8-ol as a suitable antituberculotic drug candidate for shortening the course of treatment for both active and latent form of tuberculosis. Pyridin-4-ylmethanol was involved as an analogue (derivative **3n**) and metabolic precursor of isonicotinic acid present in INH. Another interesting example of bioactive phenol is triclosan (also called irgasan, 5-chloro-2-(2,4-dichlorophenoxy)phenol; precursor of **3o**), as a widely used broad-spectrum biocidal agent and confirmed enoyl-ACP reductase inhibitor that has been highly modified, leading to many compounds with significant antimicrobial (including antimycobacterial) activity [29]. A study of Zimmermann and Curtis [30] provided a new perspective on clinically used antipyretics. Interestingly, some common antipyretics such as acetaminophen (paracetamol; ester **3p**) are able to inhibit or promote bacterial growth, affect biofilm production, affect the motility, adherence, and metabolism of pathogens, and induce or reduce the frequency of mutations, leading to antimicrobial resistance. Targeting these factors using a synthetic combination of antituberculotics and antipyretics may provide important insights for the future search of new antitubercular molecules. From the point of view of anti-TB action, it has also proved advantageous to use phenol isostere. Thiophenols (here used for thioesters **3r** and **3s**) are frequently used in the manufacture of antibiotic drugs, such as sulphonamides. Antifungals butoconazole and thiomersal are also thiophenolic derivatives [31]. Certain improvement in the profile of a potential antituberculotic drug following the incorporation of the thiophenol-based fragment has been described by Mewada et al. [32]. In addition to phenol derivatives, other bioactive compounds with the OH group have been investigated for their potential antituberculotic properties. Metronidazole (precursor of **3q**) is a synthetic drug from the group of nitroimidazoles. It is frequently used to treat protozoal and anaerobic infections. It has been demonstrated that this drug is able to suppress nonreplicating/latent mycobacterial population [33]. The efficacy against *Mtb*. under anaerobic conditions in vivo has been confirmed by Carroll et al. [34]; their study showed some effectiveness of orally administered metronidazole in terms of faster negative sputum cultivation.

### 3.2. Chemistry

**Figure 1:**
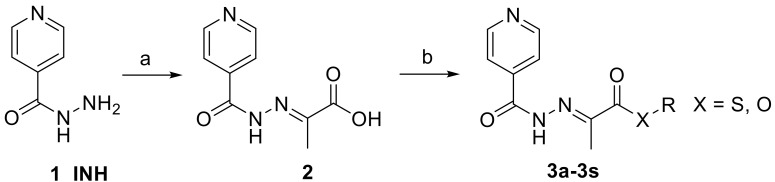
Synthesis of (*E*)-2-(2-isonicotinoylhydrazineylidene)propanoic acid esters **3** (reagents and conditions: (**a**) pyruvic acid, MeOH, reflux, 2 h; (**b**) R-OH or R-SH, *N*-(3-dimethylaminopropyl)-*N*′-ethylcarbodiimide hydrochloride, 4-(dimethylamino)pyridine, CH_2_Cl_2_, 12 h, 0 °C-rt).

(*E*)-2-(2-Isonicotinoylhydrazineylidene)propanoic acid **2** was synthesized from low-cost and commercially available isoniazid **1** and pyruvic acid in boiling methanol, according to the previously described method [14,19] with almost quantitative yield (Figure 1). The crystals of the product precipitated from the reaction mixture immediately after addition of pyruvic acid. The precipitate was washed repeatedly with cold methanol. Only the more thermodynamically stable (*E*)-isomer was detected.

(*E*)-2-(2-Isonicotinoylhydrazineylidene)propanoic acid **2** was esterified with various (thio)alcohols, or (thio)phenols by Steglich procedure employing *N*-(3-dimethylaminopropyl)-*N*′-ethylcarbodiimide hydrochloride as a coupling agent and 4-(dimethylamino)pyridine as a catalyst. Dichloromethane was used as the solvent (Figure 1). This established method is efficient and features mild conditions.The resulting urea byproduct was removed by extraction to water and organic layer was extracted successivelly with sodium bicarbonate and saturated brine and evaporated under reduced pressure. All crude products were purified by crystallization or column chromatography.

The yields of **3** ranged from 36 to 92% depending mainly on purification process. In some cases, the crystallization process had to be repeated several times, resulting in diminished yields. A relatively low yield (36%) was obtained for the triclosan-based compound **3o**, most likely due to a strong steric hindrance of the reacting hydroxyl. In addition, the crude product had to be purified several times by recrystallization. Better reaction conversions provided reactions with aliphatic substrates leading to (thio)esters **3a** and **3r**; these compounds did not need to be purified more than once. Unsubstituted phenol and naphthols (**3b** and **3i**, **3j**) or some phenols substituted by electron-donating substituents (**3c**, **3k**, **3l**) also gave good yields (52–81%). Compounds **3k** and **3l** are positional isomers; compound **3l** was prepared in lower yield (52%), most likely due to a steric hindrance. However, the yields of compounds **3i**, **3j**, **3k** and **3l** were reduced by their repeated column purification of the crude product. We also observed a partial decomposition of compounds **3i**, **3j**, **3k** and **3l** upon contact with silica gel, even after its basification using triethylamine. This phenomenon was partially eliminated in these compounds by the aprotic composition of the mobile phase (S2). The synthesis of derivative **3s** showed better reaction conversion due to the higher nucleophilicity of thiophenol compared to the synthesis of the phenol derivative **3b**; a similar result was observed in the case of compounds **3r** and **3a**.

The formation of two geometric isomers was observed in the synthesis of compounds **3i** and **3p**. In the first case, the (*Z*)-isomer, identified by NMR, was not isolated because it was obtained in a very small amount and removed during purification. Compound **3p** was prepared as a mixture of isomers (4:1 according to NMR) described below. 

Each prepared compound was characterized by ^1^H and ^13^C NMR, IR spectra and melting point. The purity was assessed by TLC and elemental analysis.

### 3.3. Microbiology

#### 3.3.1. Antimycobacterial Activity

Initially, we evaluated the esters **3** in vitro for their activity against following mycobacterial strains in Šula’s medium: fully susceptible *Mtb*. strain (331/88, i.e., H_37_Rv), drug-resistant *Mycobacterium avium* 330/88 and a clinical isolate of *M. kansasii* 6509/96. INH **1** was used as a reference compound (Table 1).

Importantly, all the esters **3** exhibited potent antimycobacterial action, especially for *Mtb*. with MIC values from ≤0.125 to 1 µM for 4-chlorophenyl (**3g**) and triclosan (**3o**) esters, followed by phenyl (**3c**) and 4-bromophenyl (**3h**) derivatives. The pyridyl ester **3n** showed the highest (but still low) MIC (0.5–1 µM). There was no sharp difference in the activity among particular (thio)esters, aromatic and aliphatic derivatives, or the corresponding isomers (**3i** and **3j**, **3k** and **3l**).

The large majority of the (thio)esters **3** exhibited better antitubercular activity than parent acid **2**, with an exception of compound **3n**. Thus, converting free carboxyl to its more lipophilic analogues is beneficial in terms of enhanced antimycobacterial properties (up to ≥8×). Importantly, identical conclusions can also be made for parent INH **1**.

*M. kansasii* 6509/96 was the second most susceptible strain (MIC of 0.25–16 µM). The best in vitro inhibition was found for both naphthyl esters (**3i**, **3j**) and thymol derivative **3l** (exceptionally low MIC values of ≤0.5 µM). Interestingly, its carvacrol-based isomer **3k** produced 64× lower activity. Additionally, thioesters **3r** and **3s** showed comparatively lower MIC. The values of simply substituted phenyl esters were similar, favouring 4-PhO and 4-F derivatives **3e** and **3f**. On the contrary, esters of 4-bromophenol, pyridin-4-ylmethanol and paracetamol (**3h**, **3n**, and **3p**, respectively) led to the highest MIC (8–16 µM). Additionally, for this strain, a wide range of the derivatives was superior to parent INH (**1**) and the acid **2**; the remaining ones produced equal MIC.

In general, polyresistant *M. avium* strain exhibited lower susceptibility (MIC from 32 µM). However, several derivatives were significantly more active than INH (**1**) and the acid **2**, especially esters of quinolin-8-ol **3m** and triclosan **3o** (32–64 µM), followed by thymol **3k**, naphthols (**3i**, **3j**) and thiols (**3r**, **3s**). However, a large majority of the derivatives showed detectable growth inhibition.

Additionally, we evaluated antimycobacterial activity of the corresponding (thio)alcohols and phenols used for the preparation of esters **3** (Table 2). The activities of phenol and its methyl, methoxy and halogen derivatives were negligible as well as for paracetamol and metronidazole (MIC ≥1000 µM). Both naphthols were more active (250–1000 µM). Terpenes exhibited MIC values of 64–500 µM, while thymol showed a mild superiority to isomeric carvacrol. Quinolin-8-ol was identified to be the most potent against all three strains, closely followed by triclosan and then 4-phenoxyphenol with lower inhibition of *M. avium*, especially. These results confirmed justification of the esters design and mutual drugs concept used in this study. To highlight connection of two bioactive scaffold in one molecules entity, it is necessary to remark that the activity of esters is usually markedly higher than in the case of both components separately, thus indicating additivity and/or synergism after their conjugation. 

Having results of initial antimycobacterial screening in our hands, we selected some esters against six MDR- and one XDR-TB strains with different resistance profiles. Thus, we selected the most anti-TB active esters (i.e., with both MIC of <0.5 µM) covering various representative types of (thio)phenolic derivatives (**3b**, **3d**, **3g**, **3i**–**3l**, **3o** and **3r**) and esters of established drugs (**3k**–**3m**, **3o**–**3q**).

Importantly, the esters were able to inhibit the growth of resistant strains, albeit with higher MIC values of 8–125 µM (Table 3). The susceptibility profile of all esters is independent on the resistance pattern; Praha 1 strain was the least sensitive. However, the results indicate a cross-resistance to the parent INH. Quinolin-8-yl ester **3m** was the most active derivative with uniform MIC of 8 µM corresponding with its MIC for *Mtb*. H_37_Rv, thus suggesting adjunctive mechanism(s) of action in addition to an “isoniazid-like” one. For example, quinolin-8-ol has been identified as a metal chelator [28]. Other more potent esters were 2-isopropyl-5-methylphenyl (i.e., derivative of thymol, **3l**), phenyl **3b** and especially 4-methoxyphenyl **3d** and 4-chlorophenyl **3g** esters, thus highlighting importance of the phenyl ring substitution by a small lipophilic group.

In these types of compounds, the highly active INH-based scaffold can eliminate INH-susceptible mycobacteria at very low concentrations, and, in the case of resistance, the pathogen is killed by the second scaffold (quinolin-8-ol, thymol, etc.). Additivity or synergism are also conceivable. 

The esters reported here are more active than recently reported substituted *N*-phenyl-2-(2-isonicotinoylhydrazineylidene)propanamides [14].

#### 3.3.2. Investigation of Mechanism of Action

The highly active esters **3** (obtained from thymol, quinolin-8-ol and triclosan, **3l**, **3m** and **3o**, respectively) were used for experimental evaluation of their mechanism of action, especially with respect to their intrinsic antimycobacterial activity. Keeping in mind their design and retained INH scaffold, we considered enoyl-ACP reductase (InhA) as a potential main target. That is why we determined MIC values of these selected esters for *Mtb*. H_37_Ra overproducing InhA together with their activity against KatG overproducing *Mtb*. H_37_Ra strain. The gene *katG* used for this construct was of *Mycobacterium smegmatis* origin.

To confirm identical mechanism of action with INH (InhA inhibition), the mycobacterial strain overproducing InhA as the target molecule should be significantly more resistant to the action of the esters due to an excess of the cellular target. On the other hand, the strain overproducing KatG activator should be more susceptible due to the facilitated bioactivation of prodrugs into the active form.

The MIC found are reported in Table 4. It covers results for H_37_Rv and all H_37_Ra strains involved in this study, i.e., those without additional genes but carrying an empty vector (pMV261, pVV16), strain overproducing InhA (pMV261-*inhA*) and strain with KatG overproduction (pVV16-*katG*). Obviously, the *Mtb*. H_37_Ra overproducing InhA_tb_ cells are more resistant to the investigated esters **3** (0.25 vs. 5 µM for **3l** and **3m**, 0.325 vs. 6 µM for **3o**). Thus, we experimentally confirmed that these compounds inhibit InhA protein as their main target. In addition, the strain overproducing KatG_smeg_ exhibited significantly lower MIC values than the control strain, especially for thymol derivative **3l**, i.e., confirming enhanced mycobacterial inhibition depending on facilitated activation mediated by KatG analogously to our previously reported 2-(2-isonicotinoylhydrazineylidene)propanamides [14].

The esters **3m** and **3o** also exhibited inhibition of INH-resistant strain of *M. avium*. This nontuberculous species lacks the enzyme KatG [35]; therefore, this activity must be due to a facilitated nonenzymatic release of isonicotinoyl radical and/or the effect of the second component of esters. It has been described that convenient substitution on hydrazide *N*^2^ can enhance its activation to promote its antimycobacterial properties [10,35]. This phenomenon can explain lower MIC values in comparison with the parent INH. An easier conversion to active form also offers a potential advantage for inhibiting strains with *katG* mutations that prevent INH bioactivation. The dual antimycobacterial activity, dependent on (thio)alcohol/phenol compounds used for esterification, is another advantage overcoming resistance to INH. 

**Figure 2:**
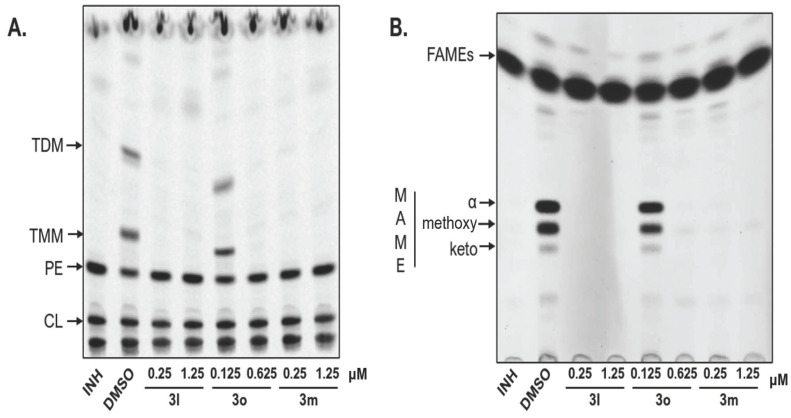
Autoradiogram of TLC analysis of (**A**) lipids and (**B**) fatty and mycolic acids methyl esters from ^14^C labelled cells of *Mtb*. H_37_Ra treated with tested compounds or DMSO or INH as a control. Lipids were separated in chloroform/methanol/water (20:4:0.5) and different forms of methyl esters were separated in *n-*hexane/ethyl acetate (95:5; 3 runs). INH: isoniazid; DMSO: dimethyl sulphoxide; TDM: trehalose dimycolates; TMM: trehalose monomycolates; PE: phosphatidylethanolamine; CL: cardiolipin; FAME: fatty acid methyl esters; MAME alpha-, methoxy- and keto- refer to the different forms of mycolic acids methyl esters.

To confirm inhibition of mycolic acid synthesis by the investigated esters **3**, *Mtb*. H_37_Ra cells were treated with different concentrations of the compounds and metabolically labelled with ^14^C acetate to monitor the synthesis of mycolic acids and lipids. Lipid analysis revealed that the treatment with esters **3** resulted in the specifically decreased production of trehalose monomycolates (TMM) and trehalose dimycolates (TDM; Figure 2A). Analysis of fatty acids isolated from whole cells confirmed the reduced synthesis of all forms of mycolic acids in contrast to the synthesis of standard fatty acids that were not affected (Figure 2B). These findings confirm the ability of the novel esters **3** to inhibit the production of mycolic acids in mycobacterial cells and thus definitely indicate InhA enzyme as their primary molecular target.

#### 3.3.3. Cytotoxicity and Selectivity

All the (thio)esters **3** and their precursors were also screened for in vitro toxicity on the standard liver cancer cell line HepG2. The assay used (CellTiter 96) is based on the reduction of tetrazolium compound MTS in living cells to formazan dye, which is determined spectrophotometrically. The decline in formazan production is due to a decrease in number of viable cells.

**Figure 3:**
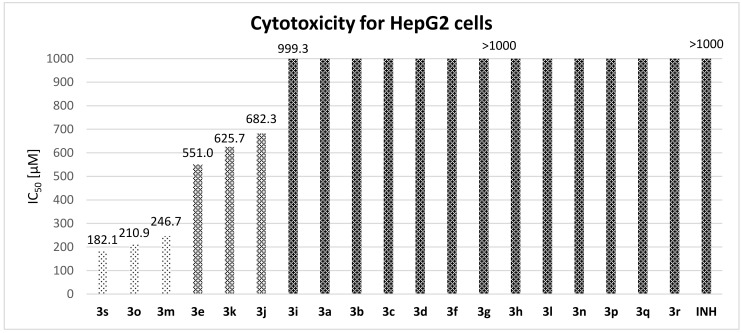
IC_50_ values for HepG2 cells.

Cytotoxicity was quantified using the standard parameter IC_50_ (Figure 3). We employed concentration range of 1–1000 μM without any solubility problem, even for highly lipophilic derivatives **3e** and **3o**.

In general, the derivatives showed minimal toxicity to eukaryotic cells. Twelve compounds avoided any cytotoxic effect at the concentration of 1000 µM (**3a**–**3d**, **3f**–**3h**, **3l**, **3n**, **3p**–**3r**); the other three esters showed IC_50_ values higher than 500 µM (4-phenoxyphenol derivative **3e**, naphthyl esters **3i** and **3j** and thymol analogue **3k**). Only three compounds (**3m**, **3o** and **3s**) were slightly more toxic (IC_50_ of 182.1–246.7 µM); thiophenyl ester **3s** was identified as the compound with the highest toxicity, while its *S*-ethyl analogue was inactive. Increased lipophilicity is generally translated into enhanced toxicity (**3e**, **3i**, **3j** and **3o**, but not in the case of **3k**). Interestingly, there was a difference between positional isomers: thymol derivative **3k** was more toxic than carvacrol **3l** and 1-naphthyl **3i** less than 2-naphtyl **3j**.

With a focus on selectivity expressed by indexes SI (defined as IC_50_/MIC; Table 5), all compounds showed securely high values for both *Mtb*. H_37_Rv and *M. kansasii* (SI of ≥493 and ≥26.4, respectively). Due to substantially negligible toxicity, some of the compounds were also selective for MDR and XDR strains despite their lower activity.

In sum, the presented esters **3** are nontoxic for liver cells that are target tissue for INH-related toxicity [36], and highly selective for mycobacteria. 

## 4. Conclusions

By modifying the established antitubercular isoniazid scaffold, we have uniquely combined this molecule with other bioactive compounds (some of which have their own antimycobacterial properties) and their analogues containing hydroxyl and thiol groups to prepare mutually active derivatives. The molecules were linked with bifunctional pyruvic acid to form hydrazone and ester bonds. In addition to dual activity, the esters are more lipophilic when compared to the parent drug, which facilitates crossing through biological barriers, including the mycobacterial cell wall.

We have synthesized nineteen esters by coupling (*E*)-2-(2-isonicotinoylhydrazineylidene)propanoic acid activated by EDC/DMAP with different bioactive alcohols, phenols, and thiols in moderate to very good yields. The esters exhibited outstanding antimycobacterial activity against *Mtb*. H_37_Rv and *M. kansasii*. Some derivatives were also unusually active against highly resistant *M. avium*. The effect on MDR and XDR-TB strains was comparatively lower, but some compounds were still active depending on the second component favouring 4-chlorophenol (**3g**) and quinolin-8-ol (**3m**). It indicates an additional mechanism of action other than inhibition of InhA that was experimentally confirmed. Due to the presence of INH scaffold, the esters selectively abolished biosynthesis of all types of mycolic acids.

Hepatotoxicity is a common side effect of antimycobacterial drugs, so we evaluated toxicity of the esters to HepG2 cells. Both IC_50_ and SI values indicate low toxicity and sufficient selectivity of examined compounds due to negligible cytotoxicity.

Taken mutually and together with their improved physicochemical properties, (*E*)-2-(2-isonicotinoylhydrazineylidene)propanoates presented here are promising hybrid antimycobacterial derivatives in terms of lower MIC, broad spectrum of activity, limited toxicity, and dual activity depending on both bioactive components.

## Figures and Tables

**Table 1:** Structure and antimycobacterial activity of esters **3**.

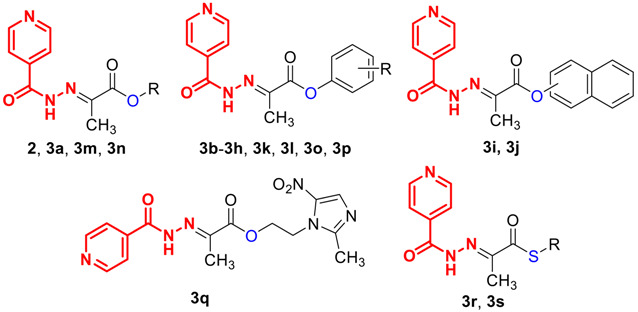									
MIC (µM)									
Code	R	*Mtb*. 331/88		*M. avium* 330/88		*M. kansasii* 6509/96			Clog*P*
		14 d	21 d	14 d	21 d	7 d	14 d	21 d	
**3a**	Me	**0.25**	**0.25**	500	1000	**2**	4	8	0.581
**3b**	H	**0.125**	**0.25**	>250	>250	8	8	8	2.016
**3c**	4-Me	**≤0.25**	0.5	>250	>250	8	8	8	2.515
**3d**	4-MeO	**0.25**	**0.25**	500	1000	4	8	8	1.935
**3e**	4-PhO	**≤0.125**	0.5	250	500	**2**	4	4	4.114
**3f**	4-F	**0.25**	0.5	500	500	**2**	4	4	2.159
**3g**	4-Cl	**≤0.125**	**≤0.125**	500	1000	4	8	16	2.729
**3h**	4-Br	**≤0.125**	**0.25**	250	500	8	8	16	2.879
**3i**	1-naphtyl	**0.25**	**0.25**	125	250	**0.25**	**0.25**	**0.25**	3.190
**3j**	2-naphtyl	**0.25**	**0.25**	125	250	**0.25**	**0.25**	**0.5**	3.190
**3k**	2-*i*Pr-5-Me	**0.25**	**0.25**	500	1000	8	16	16	3.942
**3l**	2-Me-5-*i*Pr	**0.25**	**0.25**	125	250	**0.25**	**0.25**	**0.25**	3.942
**3m**	quinolin-8-yl	**0.25**	0.5	**32**	**64**	**2**	4	4	1.903
**3n**	pyridin-4-ylmethyl	0.5	1	≥1000	≥1000	8	16	16	0.852
**3o**	5-Cl-2-(2,4-diCl-PhO)	**≤0.125**	**≤0.125**	**64**	**64**	4	4	8	6.023
**3p**	4-Ac-NH	**0.25**	0.5	1000	1000	8	16	16	1.035
**3q**	-	**0.25**	0.5	500	1000	**2**	4	4	0.821
**3r**	Et	**0.25**	**0.25**	250	500	**1**	**2**	4	1.610
**3s**	Ph	**0.25**	**0.25**	250	250	**1**	**2**	4	2.516
**2** [14]	H	1	1	500	>1000	8	16	16	−0.175
**INH 1**	-	1	1	>250	>250	8	8	16	−0.668

The lowest MIC value(s) for each strain is given in bold; INH: isoniazid.

**Table 2:** Antimycobacterial activity of precursors.

Precursor	MIC (µM)						
	*Mtb*. 331/88		*M. avium* 330/88		*M. kansasii* 6509/96		
	14 d	21 d	14 d	21 d	7 d	14 d	21 d
Phenols	≥1000	≥1000	≥1000	≥1000	≥1000	≥1000	≥1000
4-phenoxyphenol	**64**	**64**	250	500	**32**	**64**	125
1-naphthol	250	500	500	500	125	250	500
2-naphthol	500	500	1000	1000	250	500	500
Carvacrol	125	250	250	500	125	250	500
Thymol	**64**	125	250	500	125	250	500
Quinolin-8-ol	**8**	**8**	**64**	**64**	**16**	**32**	**32**
Triclosan	**32**	**32**	**64**	**64**	**16**	**32**	**32**
Paracetamol	>1000	>1000	>1000	>1000	1000	>1000	>1000
Metronidazole	>1000	>1000	>1000	>1000	1000	>1000	>1000
INH **1**	1	1	>250	>250	4	8	8

The lowest MIC value(s) for each strain is given in bold; INH: isoniazid.

**Table 3:** Activity of **3** against drug-resistant TB.

Code	MIC (µM)													
	*Mtb*. Praha 1		*Mtb*. Praha 4		*Mtb*. Praha 131		*Mtb*. 234/2005		*Mtb*. 9449/2007		*Mtb*. 7357/1998		*Mtb*. 8666/2010	
	14 d	21 d	14 d	21 d	14 d	21 d	14 d	21 d	14 d	21 d	14 d	21 d	14 d	21 d
**3b**	**16**	**16**	**16**	**16**	**16**	**16**	32	32	32	32	**16**	**16**	**16**	**16**
**3d**	**8**	**16**	**16**	32	**8**	**16**	**16**	32	**16**	**16**	**16**	**16**	**8**	**16**
**3g**	**8**	**8**	**16**	**16**	**8**	**8**	**16**	32	**16**	**16**	**16**	**16**	**8**	**16**
**3i**	64	125	**16**	32	32	32	**16**	32	32	32	**16**	32	**16**	**16**
**3j**	125	125	32	32	32	32	**16**	32	32	32	32	32	**16**	32
**3k**	125	125	**16**	32	32	64	32	32	32	32	32	32	**16**	32
**3l**	32	64	**8**	**16**	32	64	**16**	32	**16**	32	**16**	32	**16**	**16**
**3m**	**8**	**8**	**8**	**8**	**8**	**8**	**8**	**8**	**8**	**8**	**8**	**8**	**8**	**8**
**3o**	125	125	32	32	32	32	32	32	32	32	32	32	32	32
**3p**	125	125	32	32	32	64	32	64	32	64	**16**	32	32	32
**3q**	125	125	32	32	**16**	32	32	32	32	32	**16**	32	**16**	**16**
**3s**	64	125	32	64	32	64	32	64	64	64	32	64	32	32

The lowest MIC value(s) for each strain is given in bold. MDR-TB strains: Praha 1 resistant to INH, rifamycines, streptomycin (STM), ethambutol (EMB) and clofazimine; Praha 4 resistant to INH, rifamycines, STM, EMB, ofloxacin (OFX) and clofazimine; 234/2005 resistant to INH, rifamycines, STM and EMB; 9449/2006 is resistant to INH, rifamycines and STM; 7357/1998 resistant to INH, rifamycines, STM, EMB and OFX; 8666/2010 resistant to INH, rifamycines, STM, EMB, OFX and clofazimine. XDR-TB strain: Praha 131 resistant to INH, rifamycines, STM, EMB, OFX, amikacin and gentamicin.

**Table 4:** Sensitivity testing of *Mtb*. H_37_Ra overproducing InhA_tb_ or KatG_smeg_ and H_37_Rv to the selected esters **3**.

*Mtb*. Strains	MIC (μM)		
	3l	3m	3o
H_37_Rv	0.25	0.25	0.125
H_37_Ra pMV261/pVV16	0.25	0.25	0.325
H_37_Ra pMV261-*inhA*	5	5	6.25
H_37_Ra pVV16-*katG_smeg_*	0.05–0.125	0.125	0.125

*M. tuberculosis* H_37_Ra: pMV261/pVV16 strains are with no enzyme overproduction; pMV261-*inhA*: strain overproducing InhA; pVV16-*katG_smeg_*: strain overproducing KatG.

**Table 5:** Selectivity of the tested substances **3** for HepG2 cells.

Code	SI for *Mtb*. 331/88	SI for *M. kansasii* 6509/96	Code	SI for *Mtb*. 331/88	SI for *M. kansasii* 6509/96
**3a**	≥4000	≥125	**3k**	1251	39.1
**3b**	≥4000	≥125	**3l**	>4000	>4000
**3c**	≥2000	≥125	**3m**	≥493	≥61.7
**3d**	≥4000	≥125	**3n**	≥1000	≥62.5
**3e**	≥1102	≥137.8	**3o**	1687	≥26.4
**3f**	≥2000	≥250	**3p**	˃2000	≥62.5
**3g**	≥8000	≥62.5	**3q**	≥2000	≥250
**3h**	≥4000	≥62.5	**3r**	4000	≥250
**3i**	3997	3997	**3s**	728	≥45.5
**3j**	2729	2729	**INH 1**	>1000	≥62.5

## Data Availability

Data is contained within the article.
